# Thyroid Insult: Flame Retardants Linked to Alterations in Pregnant Women’s TSH Levels

**DOI:** 10.1289/ehp.118-a445b

**Published:** 2010-10

**Authors:** Kellyn S. Betts

**Affiliations:** **Kellyn S. Betts** has written about environmental contaminants, hazards, and technology for solving environmental problems for publications including *EHP* and *Environmental Science & Technology* for more than a dozen years

Polybrominated diphenyl ether (PBDE) flame retardants have been added to a wide variety of U.S. consumer goods including automobiles, airplanes, electronics, home furnishings, and furniture. Although two forms of PBDEs were phased out of use by 2004, data from the Centers for Disease Control and Prevention suggest exposure to these persistent, bioaccumulative, potentially toxic compounds remains widespread among the general U.S. population. New research now links higher exposure to PBDEs with reduced levels of thyroid-stimulating hormone (TSH) and higher rates of subclinical hyperthyroidism in women [*EHP* 118(10):1444–1449; Chevrier et al.]. The findings may have implications for maternal health and fetal development.

Thyroid hormones are known to play an essential role in fetal brain development, and altered maternal thyroid hormone levels may adversely affect child neurodevelopment. The authors cite multiple animal studies showing that PBDEs alter pregnant rodents’ thyroid functioning. In the last year, laboratory tests using human cells suggested that PBDEs may affect brain development and linked the alterations to disruptions in thyroid hormone signaling. Recent research also has linked higher PBDE exposure to reduced intelligence in children.

The authors believe their new study to be the largest to analyze associations between PBDEs and thyroid hormone during pregnancy. The 270 study participants were pregnant in 1999 and 2000 when they were recruited for the Center for Health Assessment of Mothers and Children of Salinas (CHAMACOS) study. The research team measured the women’s thyroid hormone levels, then evaluated the data with models that adjusted for potential confounding factors, such as demographic characteristics and exposure to other persistent, bioaccumulative, and toxic compounds.

Women with higher blood PBDE levels had lower levels of TSH. The women with the highest levels of PBDEs were more likely to have lower TSH but normal levels of the thyroid hormone thyroxine (T_4_), suggesting subclinical hyperthyroidism. The combination of lower TSH and “normal” T_4_—as defined by the reference range applied to lab results—could mean an individual’s T_4_ levels actually are elevated above her body’s “set point” (or optimal level) of T_4_, which varies from person to person.

Most human studies in this area have associated increased exposure to PBDEs with elevated free T_4_. The only other study known to examine PDBEs and thyroid hormone specifically in pregnant women—which included only nine women—did not find increased T_4_, but the authors did not examine TSH levels. In contrast, other studies of pregnant women’s thyroid hormone levels have linked higher exposure to organochlorine pesticides and polychlorinated biphenyls—which have chemical structures and properties similar to those of PBDEs—with increased TSH and decreased T_4_.

